# Fetal Growth Restriction, Autism Spectrum Disorder and Attention-Deficit/Hyperactivity Disorder-Connecting the Dots: A Narrative Review

**DOI:** 10.3390/children13010009

**Published:** 2025-12-19

**Authors:** Alexios Kozonis, Maria Papadoliopoulou, Ioannis Margaris

**Affiliations:** 1 3rd Department of Obstetrics and Gynaecology, Attikon University Hospital, Medical School, National and Kapodistrian University of Athens, 1 Rimini Street, 12462 Athens, Greece; 2 4th Department of Surgery, Attikon University Hospital, Medical School, National and Kapodistrian University of Athens, 1 Rimini Street, 12462 Athens, Greece

**Keywords:** fetal growth restriction (FGR), autism spectrum disorder (ASD), attention-deficit/hyperactivity disorder (ADHD)

## Abstract

Background/Objectives: Fetal growth restriction (FGR), formerly known as intrauterine growth retardation (IUGR), is defined as a fetus’ failure to reach its genetically predetermined growth potential. FGR has also been implicated in the development of autism spectrum disorders (ASD) and attention-deficit/hyperactivity disorder (ADHD), though strong supporting literature has yet to be published. This study aims to review the existing associations between FGR and autism spectrum disorder or attention-deficit hyperactivity disorder as studied in relevant literature, as well as the mechanisms that provide explanation of that association. Methods: We used a combination of the terms ‘Autism spectrum disorder’ OR ‘Attention deficit hyperactivity disorder’ OR ‘neurodevelopmental disorders’ AND ‘intrauterine growth retardation (IUGR)’ OR ‘fetal growth restriction (FGR)’ in an electronic search of PubMed/MEDLINE and Scopus databases. Results: After evaluating the existing literature, we found only a few studies assessed the risk of developing ASD or ADHD in IUGR/FGR children. Neurodevelopmental disorders have generally been linked to very low birth weight, small for gestational age neonates (SGA), prematurity, somatic mutations, and intrauterine caffeine and alcohol exposure. While available evidence supports the notion that IUGR/FGR is related to cognitive impairment and behavioural disorders, the association with ASD or ADHD remains elusive due to the marked variability in the reported outcomes. Few studies have reported a respective higher risk for autism spectrum disorders, yet most of them have failed to identify a statistically significant correlation. Conclusions: While autism spectrum disorders and attention deficiency disorder have been generally associated with FGR children, the existing body of literature offers limited evidence to support this theory.

## 1. Introduction

Fetal Growth Restriction (FGR), formerly known as intrauterine growth retardation (IUGR), is defined as a fetus’ failure to reach its genetically predetermined growth potential, usually identified as (a) an estimated fetal weight or abdominal circumference below the 3rd percentile; or (b) an estimated fetal weight or abdominal circumference below the 10th percentile in combination with abnormal fetal Doppler velocimetry; or (c) a significant decline in fetal growth on serial assessments [1]. FGR is typically diagnosed via ultrasound, particularly when fetal biometric parameters fall below expected norms [1]. The condition affects approximately 5–10% of pregnancies and has a multifactorial etiology, including fetal, maternal, and uteroplacental causes [2,3]. It can be symmetrical or asymmetrical, late or early (32 weeks of gestation), and it predisposes to severe perinatal morbidity and possibly mortality. On the contrary, the term small for gestational age (SGA) does not refer completely to FGR neonates, as SGA refers to neonates born with a birthweight less than the 10th percentile. Those two conditions—which are similar, but not identical—have been associated with severe impairment of fetal brain development, consequently leading to neurobehavioral, motor, and cognitive dysfunction, among other conditions [1,4].

More specifically, FGR has also been implicated in the development of two common neurodevelopmental disorders—autism spectrum disorder (ASD) and attention-deficit/hyperactivity disorder (ADHD). ASD is characterized by communication difficulties, impaired social interaction, and repetitive or unusual behaviour, and has an estimated prevalence up to 1% [5,6]. In addition, ADHD is a condition defined by inattention, hyperactivity, and impulsivity, with approximately 4% worldwide prevalence and unclear etiology [7].

FGR is believed to be associated with neurodevelopmental disorders due to mechanisms of suboptimal growth, hypoxic stress, brain sparing, etc. However, the association is yet to be confirmed because of a paucity of data in the existing literature and failure to prove the exact mechanisms and etiology behind this remark; furthermore, FGR overlaps with several maternal–fetal conditions such as pre-eclampsia, prematurity, low birth weight, etc., that are also thought to raise the probability of impaired neurodevelopment in neonates [8,9]. This study aims to review the existing associations between FGR and autism spectrum disorder or attention-deficit hyperactivity disorder as studied in the relevant literature, as well as the mechanisms that provide explanation of that association.

For the purposes of this article, and since most of the available literature was published before the 2016 consensus on FGR, both terms (FGR and IUGR) will be used interchangeably.

## 2. Materials and Methods

For the aforementioned purposes, a narrative review was conducted using studies of various complexity and design. We conducted a systematic search of the literature to identify eligible studies elaborating on the relation between FGR and autism or attention-deficit disorder using the following terms for PubMed: (All terms) (“Autism spectrum disorder” OR neurodevelopmental OR attention-deficit) AND (“Fetal growth restriction” OR IUGR). Filters: 2005–2025. Results: 699. For Scopus, the following search terms were used: (TITLE-ABS-KEY) ((“Autism spectrum disorder” OR autism OR neurodevelopmental OR attention-deficit) AND (“Fetal growth restriction” OR IUGR). Filters: 2005–2025. Results: 37).

An electronic search of PubMed/MEDLINE and Scopus was carried out between 1 July 2025 and 14 August 2025, reviewing all relevant literature published in the English language regarding intrauterine growth retardation and its association with autism spectrum disorder and attention-deficit hyperactivity disorder. We used a combination of the terms ‘Autism spectrum disorder’ OR ‘Attention-deficit hyperactivity disorder’ OR ‘neurodevelopmental disorders’ AND ‘intrauterine growth retardation (IUGR)’ OR ‘fetal growth restriction (FGR)’. Both original articles and review articles that involved patients with FGR/IUGR and an autism spectrum diagnosis or attention-deficit hyperactivity disorder diagnosis, without a publication frame, were deemed eligible. Twin studies and studies conducted in animals were excluded. All articles that studied the effect of a substance, e.g., alcohol, on neurodevelopmental disorders were also excluded. The article selection procedure and data extraction were assessed by two independent authors. Any areas of disagreement were resolved on an individual basis through a case-by-case discussion.

## 3. Results and Discussion

**Figure 1:**
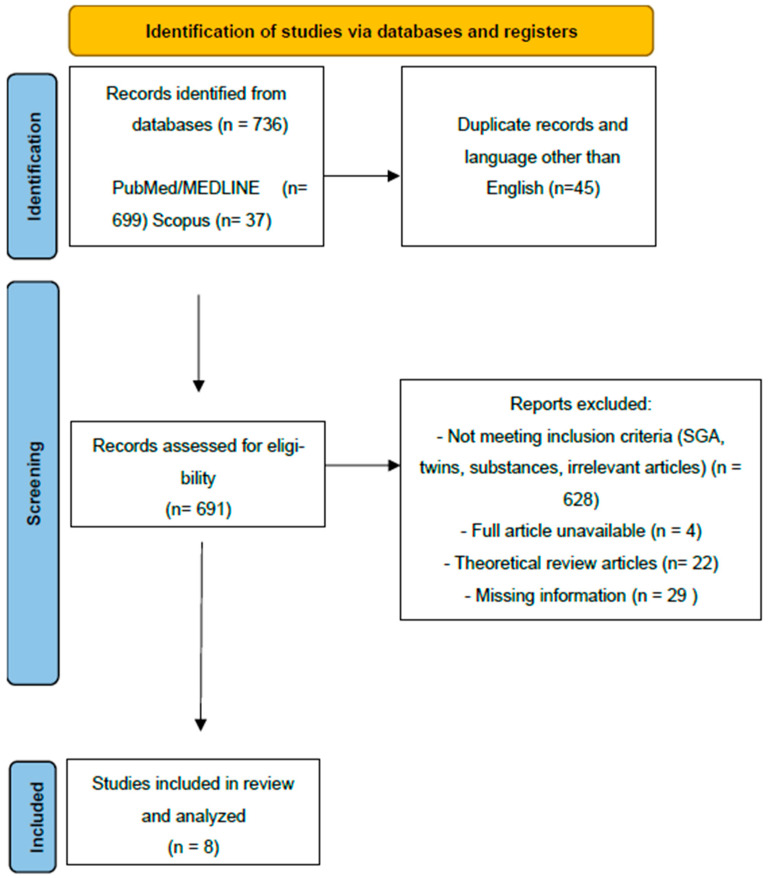
Prisma flow chart.

The study selection process is presented in Figure 1.

Briefly, 736 articles were identified through a systematic search of the PubMed/MEDLINE and Scopus databases. (Figure 1). After the removal of duplicates and all studies in languages other than English, 691 articles were screened for potential inclusion. The articles excluded and the reasons for exclusion are also presented. Ultimately, eight articles were included and underwent further analysis in this review article. All articles screened by the research team (A.K. and M.P.) were put through Covidence, where further abstract and full-text screening took place, until the final list of included articles was agreed upon by all authors. (Table 1)

Fetal growth restriction, formerly known as intrauterine growth retardation (IUGR), is defined as a fetus’ failure to reach its genetically determined growth potential. It has an incidence of up to 9% of pregnancies in high-income countries and rates can increase dramatically in low-income countries and reach almost 30%. The factors associated with FGR are fetal, maternal, and placental. Overall, the most common cause is placental insufficiency and abnormalities in the placental blood flow, resulting in limited oxygen transfer and nutrition for the fetus. FGR is typically diagnosed via ultrasound, particularly when fetal biometric parameters fall below expected norms. It can be symmetrical or asymmetrical, late or early (32 weeks of gestation), and it is thought to be associated with perinatal morbidity and mortality [1].

On the other hand, SGA refers to infants that are born with a birthweight less than the 10th percentile, likely because they are genetically small. In 2016, a consensus of FGR definition was reached and included placental dysfunction parameters and biometric measures, emphasizing that FGR refers to infants with suboptimal growth due to pathological changes during pregnancy. Those conditions, which have previously been mistaken for one another, are thought to be associated with neurodevelopment disorders, including autism spectrum disorder (ASD) and attention-deficit/hyperactivity disorder (ADHD) [1,6,7,8,9,10,11,12,13,14,15].

Autistic spectrum disorder is characterized by impaired social interactions, communication difficulties, and stereotypical behavioural patterns, affecting up to 1% of the global population [16]. One person per one thousand births will be diagnosed with autism disorder at some point in their lifetime [7,8,9,10,11,12,13,14,15,16,17]. While the etiology is thought to be genetic, environmental factors associated with autism include exposure to rubella, thalidomide, or valproate during pregnancy [16]. Until today, there has been no specific etiology for ASD. Proposed maternal conditions and perinatal factors include threatened abortion, maternal diabetes, pre-eclampsia, preterm birth, low birth weight, intrauterine growth retardation, and low Apgar scores [5].

Simultaneously, up to 75% of children with ASD are also diagnosed with intellectual disability. ASD and ID are frequently combined, but also share some manifestations. Children with these conditions present with developmental delay, abnormal verbal communication, and social difficulties.

Attention-deficit/hyperactivity disorder (ADHD) is a neurodevelopmental disorder characterized by inattention, hyperactivity, and impulsivity. Its worldwide prevalence is approximately 3.4–7.2%. Its etiology is still unknown [18].

FGR, as mentioned before, has shown increased occurrence in tandem with neurodevelopmental disorders. Suboptimal in utero growth is thought to play an important role in the development of the cardiovascular and cerebrovascular systems of the developing fetus, which continues after birth and into adulthood. Hypoxic stress can elicit many physiological responses to the developing fetus, including brain sparing, a response which aims to preserve the development of vital organs [19]. The brain sparing response includes redistribution of blood flow to essential vascular beds, e.g., the brain, heart, and adrenal glands, and decreased blood flow to non-essential vascular beds, e.g., the liver and skeletal muscle. The consequences for the fetus include, in addition to many vascular and endothelial changes, decreased head circumference and total brain weight, decreased grey and white matter volume and cell number, loss of cerebral vasoreactivity, impaired neuro-vascular units, and regional perfusion alteration [19]. A brain sparing diagnosis is established with the use of a Doppler ultrasound to visualize the flow through the middle cerebral artery (MCA) and a pulsatility index calculation which is indicative of downstream vascular resistance [20].

Initially, when brain sparing begins, blood flow preferentially supplies the frontal brain region. Later on, perfusion is shifted towards the basal ganglia, where nuclei responsible for motor control, learning, behaviours, and emotions are located.

As mentioned above, the fetus responds to hypoxia with brain sparing, i.e., vasodilation of the cerebral vasculature, in order to protect and preserve brain growth. However, it has been shown that brain sparing may lead to cerebrovascular remodelling, loss of cerebral vasoreactivity, and neurodevelopment dysfunction [21,22].

After an evaluation of existing literature, it was found that only a few studies assessed the risk of developing ASD or ADHD in FGR children.

In general, neurodevelopmental disorders have been associated with very low birth weight, SGA neonates, prematurity, somatic mutations, intrauterine caffeine and alcohol exposure, and SGA neonates, a particular population commonly mistaken as FGR neonates [23]. Variability in definitions and lack of biological mechanistic data and studies only conducted in animal models are confusing factors and weaken association strength [6,14,15,24,25,26,27,28,29,30].

While many studies examine, either retrospectively or prospectively, the neurodevelopment dysfunctions of FGR children, the results are most often focused on IQ measures or, in general, on the motor, language, and speech function of children, most of them without including a diagnosis of a certain neurodevelopmental disorder. Authors have highlighted the heterogeneity of primary outcomes and assessment tools and variability in FGR definition and the lack of a control population [31]. To our knowledge, this is the first review article examining the association between autism spectrum disorders and ADHD with FGR children.

In 1988, Matilainen et al. compared IUGR children with appropriate-for-gestational-age and full-term children by measuring their general intelligence quotient (IQ) and deficiencies in intellectual function such as perceptuomotor functions, language, and social maturity at preschool. IUGR children were found to have lower IQ scores than full-term controls and were more commonly diagnosed with developmental disabilities [32].

Inappropriate intrauterine growth was considered to be associated with intellectual disability in a retrospective study examining 2.277 Caucasian children and 348 Aboriginal children in Western Australia. In this study, infants with excessive intrauterine growth (POBW > 124 percentile) were more likely to be diagnosed with intellectual disability associated with autism spectrum disorders (odds ratio 2.36 95% CI (0.93–6.03) [33].

In a retrospective univariate and multivariate analysis of 383.153 births in Western Australia between 1984 and 1999, maternal conditions, including a poor intrauterine environment, were associated with elevated risks of intellectual disability and autism disorders with ID were associated with threatened abortion before 20 weeks of gestation and poor fetal growth [5].

Twin studies like the Roots of Autism and ADHD Twin Study in Sweden (RATSS) support birth weight effects beyond a shared genetic predisposition. The findings of that recent study suggest an association between low birth weight and neurodevelopmental conditions, but they also acknowledge the importance of genetics because the associations observed were only statically significant among monozygotic twins. Twin studies were not included in our analysis due to altered and specific factors including maternal, fetal, and placental parameters [34].

The association between IUGR, postnatal energy restriction, and neurodevelopment was examined using multivariate logistic regression techniques in a recent article by Uberos et al. [8]. IUGR was associated with behavioural disorders (OR 2.60; 95% CI 1.25, 5.40) and delayed cognitive development (OR 2.64; 95% CI 1.34, 5.20). In very-low-birth-weight (VLBW) infants, IUGR was associated with behavioural disorders. On the other hand, postnatal energy restriction was significantly associated with motor disorders, infantile cerebral palsy and sensory disorders [8].

In a 2021 retrospective cohort study in Taiwan, where all births between 2000 and 2010 were recorded and 517 cases of ASD were found, the adjusted odds ratio in IUGR children was 8.6 [13].

In a review from 2016, in which cognitive data from 15 studies and behavioural data from 6 studies were analyzed, children with IUGR and SGA had lower cognitive scores compared with controls 0.38 *p* < 0.00001, but ADHD incidence was not significantly different between the two groups [35]. Data from this particular study was not evaluated in our review due to its misconception and differentiation between IUGR neonates and SGA neonates.

A few studies showed special interest in premature IUGR. Korzeniewski et al., in a multicenter prospective observational study, evaluated 889 10-year-old children who had been born before 28 weeks of gestation for the presence of ASD. Extremely preterm infants with severe FGR were found to have an increased risk of behavioural dysfunctions, including ASD, but that did not reach statistical significance [11]. Similarly, a retrospective cohort study evaluating very preterm infants with IUGR and appropriate-for-gestational-age (AGA) infants concluded that the former were more likely to have a positive result according to the Modified Checklist for Autism (OR 2.12) [14]

A remarkable study in FGR research was a prospective multicenter unblinded randomized trial conducted from 2005 to 2010 in European centres, the Trial of Randomized Umbilical and Fetal Flow in Europe (TRUFFLE) in singleton pregnancies at 26–32 weeks of gestation with FGR diagnosis; this was the first wide study that, while monitoring management strategies in FGR children, also revealed interesting correlations after following up with these children [36]. In the study, five hundred and three women were randomized to deliver according to short-term variation and umbilical artery Doppler criteria. The purpose of this was to evaluate survival without cerebral palsy and neurosensory impairment or with a Bayley-III developmental score of < 8.5 at 2 years of age. The TRUFFLE study found neurodevelopmental impairment in 10% of the study population [37,38]. That research study continued in TRUFFLE study 2, a randomized controlled trial that compared the timing of delivery in FGR children with or without the use of the umbilical cerebral ratio (UCR) with the children’s neurodevelopmental outcomes. The study is still ongoing [38]. Due to the physiology of FGR neonates, their gestation is often complicated by prematurity. Since prematurity alone is considered to increase the probability of an ASD or ADHD diagnosis, a few studies tried to evaluate preterm neonates with and without FGR. In a 2011 study by Morsing et al., very preterm IUGR neonates had an increased risk of cognitive impairment compared to other preterm neonates. However, tests that evaluated ADHD likelihood did not differ [10]. More recently, Check et al. found significantly lower Bayley-III Scores in FGR children in a group of preterm neonates born before 30 weeks of gestation [9].

Interestingly, many studies attempted to identify an association between magnetic resonance features and neurodevelopmental disorders in FGR infants. A study found ten common resting-state networks involving cortical and subcortical regions and the cerebellum. FGR infants showed patterns of increased connectivity in the visual network and decreased connectivity in the auditory/language and dorsal attention networks. Yet no significant differences between groups were found after using a seed-based correlation analysis [12]. In a retrospective study conducted in 2019, T2-weighted images were obtained from IUGR and AGA infants. Smaller total intracranial volume and altered Jacobian determinants in anatomical regions such as the Rolandic operculum, gyrus rectus, hippocampus, thalamus, etc., were noticed in IUGR infants [14].

Some important limitations need to be mentioned. First of all, the number of included studies is relatively small and that most of them were of a retrospective design and had small sample sizes. Residual confounding, due to maternal health or various socioeconomic factors, etc., may have introduced additional bias and hampered the generalizability of the results. Additionally, there was significant heterogeneity and variability among studies with regard to the definitions and measures used to correlate IUGR/FGR and autism spectrum disorders or attention-deficit/hyperactivity disorder. Finally, the included studies lacked biological mechanistic data that could shed light on the precise underlying etiologic factors. Future large-scale prospective population studies are needed to strengthen the current level of evidence.

## 4. Conclusions

While autism spectrum disorders end attention deficiency disorder have been generally associated with FGR children, the existing body of literature offers limited evidence to support this theory. More well-designed studies are needed, including an in-depth analysis of the possible underlying mechanisms and factors responsible.

## Figures and Tables

**Table 1:** Eligible studies about FGR’s association with ASD or ADHD.

Author (Year)	Type of Study	Population	Results	Outcome
Langridge (2013) [5]	Retrospective cohort	Western Australia, all births between 1984 and 1999;	n = 383,153, ASD with ID = 727; ASD without ID =452; ID without ASD = 4576	IUGR is associated with an increased risk of ID; ASD with ID has a weak association with IUGR
Morsing (2011) [10]	Prospective study	Sweden, children born at 24–29 weeks of gestation	Preterm IUGR = 34 Preterm AGA = 34 Term AGA = 34	Very preterm IUGR children had an increased risk of cognitive impairment compared to preterm children for other reasons. Behaviour and Brown attention-deficit disorder test results did not differ
Korzeniewski (2017) [11]	Multicenter prospective observational study	889 children born before 28 weeks (evaluated at the age of 10 years)	52 severe FGR, 113 less severe FGR	Extremely preterm children with severe FGR had elevated risk of behavioural dysfunctions, including ASD (not statistically significant)
Padilla (2017) [12]	Retrospective cohort study	20 singleton preterm FGR infants; 20 preterm AGA infants; 20 term AGA infants	—	The FGR group had highest risk of early autist features (M-CHAT Modified Check list of Autism + MRI data acquisition
Lee (2021) [13]	Retrospective cohort study	Taiwan, diagnosis of ASD between 2000 and 2010 (from national database)	n = 62,051, ASD = 517	8.6 adjusted odds ratio IUGR
Sacchi (2021) [14]	Retrospective cohort study	Very preterm infants enrolled in the Evaluation of Preterm Imaging Study	n = 314, IUGR = 49	Very preterm IUGR children were more likely to have a positive Modified Checklist for Autism, OR 2.12 compared to very preterm AGA children
Uberos (2022) [8]	Retrospective cohort study	Very-low-birth-weight infants, some with IUGR	n = 337, IUGR = 64	IUGR is associated with behavioural disorder (OR 2.60; 95% CI 1.25, 5.40) and delayed cognitive development (OR 2.64; 95% CI 1.34, 5.20)
Check (2024) [9]	Retrospective cohort study	Infants born before 30 weeks’ gestation in the prospective Neonatal Neurobehavior and Outcomes in Very Preterm Infants study, 2014–2016, US	n = 529 very preterm infants; 46 had FGR	FGR was associated with significantly lower Bayley-III Scores regardless of pre-eclampsia status

Note. AGA = appropriate for gestational age; IUGR = intrauterine growth restriction; ASD = autism spectrum disorder; ID = intellectual disability; OR = odds ratio; M-CHAT = Modified Checklist for Autism in Toddlers.

## Data Availability

The original contributions presented in this study are included in the article. Further inquiries can be directed to the corresponding author.
